# The role of corticospinal and extrapyramidal pathways in motor impairment after stroke

**DOI:** 10.1093/braincomms/fcac301

**Published:** 2022-11-21

**Authors:** Theresa Paul, Matthew Cieslak, Lukas Hensel, Valerie M Wiemer, Christian Grefkes, Scott T Grafton, Gereon R Fink, Lukas J Volz

**Affiliations:** Medical Faculty, University of Cologne, and Department of Neurology, University Hospital Cologne, 50937 Cologne, Germany; Department of Psychiatry, Perelman School of Medicine, University of Pennsylvania, Philadelphia, PA 19104, United States of America; Medical Faculty, University of Cologne, and Department of Neurology, University Hospital Cologne, 50937 Cologne, Germany; Medical Faculty, University of Cologne, and Department of Neurology, University Hospital Cologne, 50937 Cologne, Germany; Medical Faculty, University of Cologne, and Department of Neurology, University Hospital Cologne, 50937 Cologne, Germany; Institute of Neuroscience and Medicine, Cognitive Neuroscience (INM-3), Research Centre Juelich, 52425 Juelich, Germany; Department of Psychological & Brain Sciences, University of California, Santa Barbara, CA 93106, United States of America; Medical Faculty, University of Cologne, and Department of Neurology, University Hospital Cologne, 50937 Cologne, Germany; Institute of Neuroscience and Medicine, Cognitive Neuroscience (INM-3), Research Centre Juelich, 52425 Juelich, Germany; Medical Faculty, University of Cologne, and Department of Neurology, University Hospital Cologne, 50937 Cologne, Germany

**Keywords:** diffusion spectrum imaging, Wallerian degeneration, recovery of function, anisotropy, structural reserve

## Abstract

Anisotropy of descending motor pathways has repeatedly been linked to the severity of motor impairment following stroke-related damage to the corticospinal tract. Despite promising findings consistently tying anisotropy of the ipsilesional corticospinal tract to motor outcome, anisotropy is not yet utilized as a biomarker for motor recovery in clinical practice as several methodological constraints hinder a conclusive understanding of degenerative processes in the ipsilesional corticospinal tract and compensatory roles of other descending motor pathways. These constraints include estimating anisotropy in voxels with multiple fibre directions, sampling biases and confounds due to ageing-related atrophy. The present study addressed these issues by combining diffusion spectrum imaging with a novel compartmentwise analysis approach differentiating voxels with one dominant fibre direction (one-directional voxels) from voxels with multiple fibre directions. Compartmentwise anisotropy for bihemispheric corticospinal and extrapyramidal tracts was compared between 25 chronic stroke patients, 22 healthy age-matched controls, and 24 healthy young controls and its associations with motor performance of the upper and lower limbs were assessed. Our results provide direct evidence for Wallerian degeneration along the entire length of the ipsilesional corticospinal tract reflected by decreased anisotropy in descending fibres compared with age-matched controls, while ageing-related atrophy was observed more ubiquitously across compartments. Anisotropy of descending ipsilesional corticospinal tract voxels showed highly robust correlations with various aspects of upper and lower limb motor impairment, highlighting the behavioural relevance of Wallerian degeneration. Moreover, anisotropy measures of two-directional voxels within bihemispheric rubrospinal and reticulospinal tracts were linked to lower limb deficits, while anisotropy of two-directional contralesional rubrospinal voxels explained gross motor performance of the affected hand. Of note, the relevant extrapyramidal structures contained fibres crossing the midline, fibres potentially mitigating output from brain stem nuclei, and fibres transferring signals between the extrapyramidal system and the cerebellum. Thus, specific parts of extrapyramidal pathways seem to compensate for impaired gross arm and leg movements incurred through stroke-related corticospinal tract lesions, while fine motor control of the paretic hand critically relies on ipsilesional corticospinal tract integrity. Importantly, our findings suggest that the extrapyramidal system may serve as a compensatory structural reserve independent of post-stroke reorganization of extrapyramidal tracts. In summary, compartment-specific anisotropy of ipsilesional corticospinal tract and extrapyramidal tracts explained distinct aspects of motor impairment, with both systems representing different pathophysiological mechanisms contributing to motor control post-stroke. Considering both systems in concert may help to develop diffusion imaging biomarkers for specific motor functions after stroke.

## Teaser

Paul *et al*. show that motor impairment post-stroke is best characterized by combining compartment-specific anisotropy of descending ipsilesional corticospinal tract fibres capturing Wallerian degeneration and extrapyramidal brainstem structures serving as compensational structural reserve.

## Graphical Abstract

**Graphical abstract:**
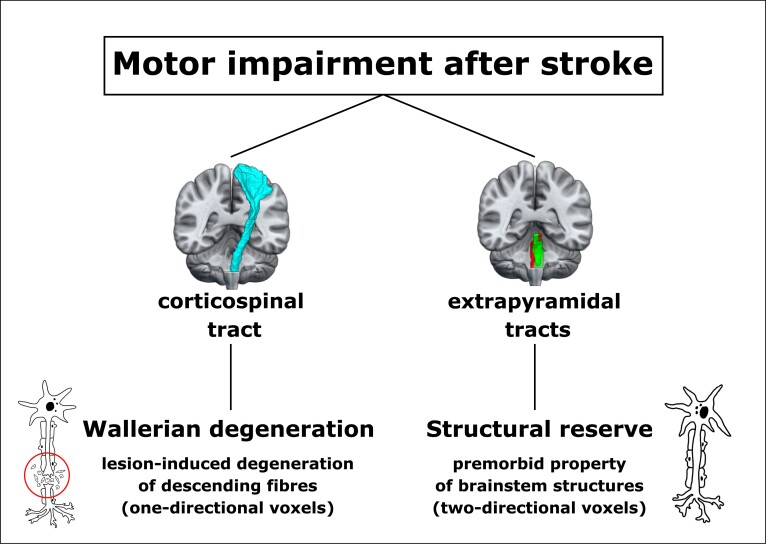

## Introduction

Stroke-related motor deficits are often caused by damage to the corticospinal tract (CST) and associated white matter (WM) changes are frequently assessed using anisotropy derived from diffusion MRI (dMRI). Studies commonly report a relationship between decreased anisotropy in various parts of the ipsilesional CST and the severity of motor impairment of the upper^1-3^ and lower limbs,^4-6^ highlighting anisotropy as a promising biomarker for motor recovery post-stroke.^3^ However, anisotropy measures have yet to find their way into clinical practice as individual predictions of motor impairment and recovery remain challenging for several reasons.

First, most studies use a single measure of motor impairment, hindering a differentiated analysis of various aspects of motor performance. Second, it remains unknown which biological processes may underlie changes in anisotropy or give rise to the correlation between CST anisotropy and motor behaviour, rendering conclusive mechanistic interpretations difficult. In line with the notion that the degree of anisotropy reduction reflects the extent of structural damage—often referred to as *microstructural integrity*—lower CST anisotropy coincides with more severe motor impairment.^1^ Given that this correlation can still be observed when calculating anisotropy without including the lesion itself, a commonly held view is that this association is driven by *Wallerian degeneration* of descending fibres, a process that describes how axons passing through the lesion degenerate over time.^7^ However, several methodological limitations hinder a confirmation of this notion as previous dMRI studies primarily relied on fractional anisotropy (FA) derived from diffusion tensor imaging (DTI). DTI cannot adequately resolve multiple fibre directions within a given voxel,^8^ yielding misleading FA estimates for multi-directional voxels entailing crossing or kissing fibres.^9,10^ Numerous studies have circumvented this issue by focusing the analyses on specific parts of the CST, which contain densely packed, parallel-running descending fibres,^11^ therefore thought to reflect the extent of Wallerian degeneration.^12^ Commonly used regions of interest (ROIs) include the pons,^13,14^ the cerebral peduncle (CP),^15,16^ or the posterior limb of the internal capsule (PLIC).^17-19^ Unfortunately, such ROI-based approaches introduce new pitfalls. First, the limited number of voxels within the typically used ROIs aggravates sampling biases.^2^ Moreover, stroke patients usually feature considerable degrees of ageing-related WM atrophy^20^ in addition to stroke-induced damage, with certain parts of the brain such as the PLIC being especially prone to ageing-related degeneration.^21,22^ Thus, ageing-related confounds might bias the quantification of Wallerian degeneration, especially when applying ROI-based approaches. Taken together, this leads to the question whether ipsilesional CST anisotropy is primarily reflective of Wallerian degeneration, which would emphasize its potential as biomarker, or whether ageing-related degeneration and methodological limitations may bias the commonly observed correlation with motor performance.

A third aspect that hinders the usage of ipsilesional CST anisotropy as a biomarker is the motor system’s ability to compensate for CST damage via alternate fibre tracts not directly affected by the lesion. Therefore, a patient may not necessarily have to rely on spared fibres of the lesioned CST alone. Specifically, non-crossing fibres of the contralesional CST^23^ or bihemispheric extrapyramidal pathways including the reticulospinal (reticuloST) and rubrospinal tract (rubroST)^1,5^ may compensate for ipsilesional CST damage by supporting basal motor skills via their projections to proximal arm and leg muscles. However, mixed findings hinder a conclusive interpretation of their role in motor control post-stroke. While some studies argue that extrapyramidal pathways successfully support gross motor performance, others ascribe a potential maladaptive influence to an upregulated extrapyramidal system, leading to a dysfunctional increase in flexor synergies.^24,25^ While an increase in anisotropy has been conceptualized as an upregulation of the extrapyramidal system caused by structural reorganization post-stroke,^26-28^ other studies could not replicate this finding, even reporting a decrease in anisotropy.^29-31^ Thus, the question arises whether the frequently observed association between extrapyramidal tract anisotropy and motor behaviour is a result of structural changes in the extrapyramidal system or whether subjects with a relatively high premorbid level of extrapyramidal anisotropy are better equipped to compensate for CST deficits through reliance on their structural reserve.^32^ Notably, the assessment of anisotropy in extrapyramidal tracts suffers from the same limitations as described above for the CST, rendering the interpretation of anisotropy in the extrapyramidal system challenging.

**Figure 1:**
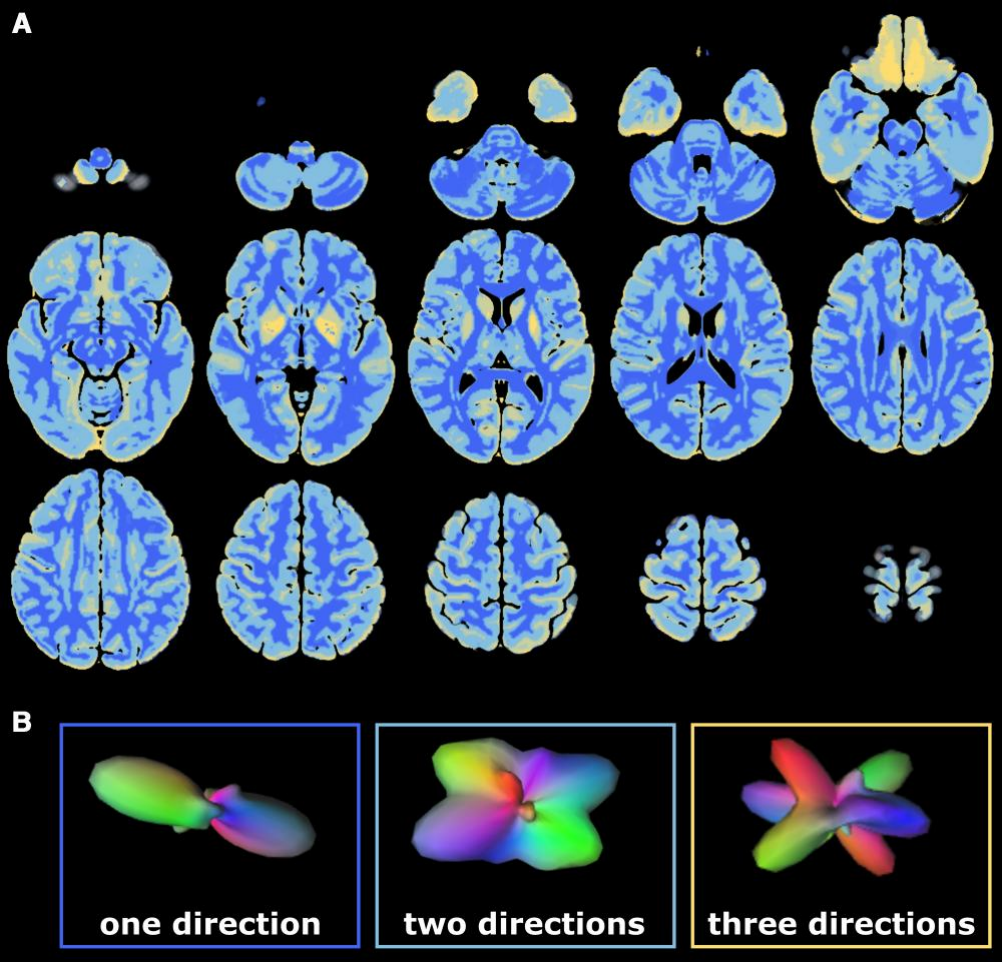
**Deterministic brain mask for whole-brain compartmentalization.** (**A**) The mask was created by Volz *et al.* based on 630 subjects from the Human Connectome Project.^10^ Each colour represents a single compartment containing voxels with a certain number of trackable directions [dark blue = one direction, light blue = two directions, yellow = three (or more) directions]. Images depicted in (**A**) were created based on the nifti-file published by Volz *et al*.^10^ (**B**) The number of trackable directions was determined based on the underlying ODF within a given voxel, which can simultaneously depict several diffusion directions. Depending on the number of peaks exceeding a certain threshold, each voxel was assigned to a specific compartment representing the number of trackable intravoxel directions. Exemplary ODFs with different numbers of peaks are shown in (**B**).

In sum, a better understanding of the mechanisms underlying degeneration of the CST and behavioural compensation of specific motor functions via alternate motor pathways is crucial to pave the way for the usage of anisotropy as a biomarker in clinical care. To address these issues, we assessed chronic stroke patients using motor tests that differentiate various aspects of motor performance and included age-matched and young controls to discern ageing- and stroke-related WM degeneration. Diffusion spectrum imaging (DSI) was employed to better resolve multiple diffusion directions per voxel.^33^ Anisotropy was conceptualized as generalized fractional anisotropy (gFA), which allowed us to quantify the magnitude of multiple intravoxel directions.^34^ Moreover, a compartmentwise analysis approach that differentiates voxels according to their number of trackable directions^10^ (cf. Fig. 1) enabled us to separately analyze voxels containing one-directional or multi-directional fibres.

By focusing on voxels containing only one dominant fibre direction, we assessed anisotropy of descending fibres along the length of the entire ipsilesional CST, overcoming the limitations of ROI-based approaches. Assuming that the correlation with motor impairment was genuinely driven by Wallerian degeneration, we hypothesized a positive correlation between motor performance and anisotropy mainly driven by one-directional CST voxels. Given the seminal role of the CST, we expected this correlation to emerge for both basal and complex motor skills of the upper and lower limbs. In line with the hypothesis that other descending motor tracts can partially compensate stroke-related deficits in gross motor control,^4,26,28,29^ we expected anisotropy within contralesional CST and extrapyramidal tracts to be linked to basal arm and leg functions. We further hypothesized a combination of alternate motor pathways and ipsilesional CST to explain more behavioural variance than the latter alone as motor performance ultimately depends on both degenerative and compensatory processes. Finally, if compensation was the result of an upregulation of the extrapyramidal system caused by structural reorganization, we would expect an increase in anisotropy in patients compared with age-matched controls, whereas no group difference would imply the reliance on a structural reserve instead.

## Materials and methods

### Subjects

**Figure 2:**
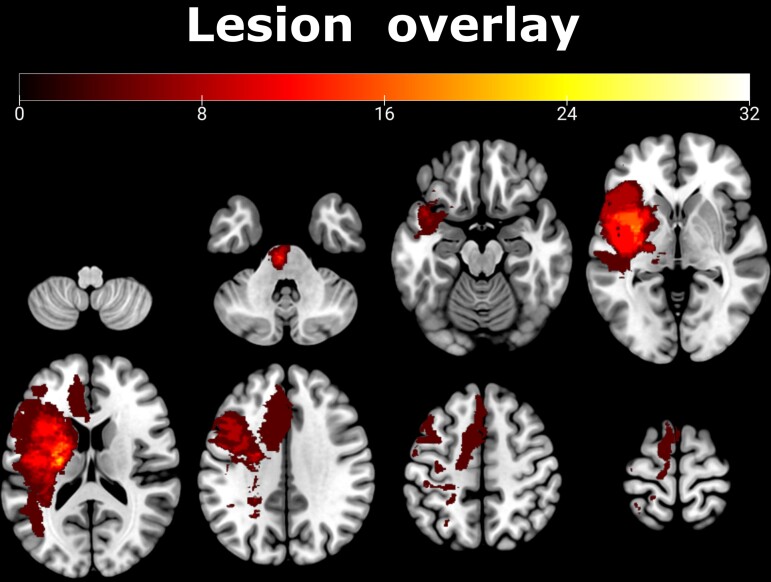
**Lesion overlay showing affected voxels across all 25 patients in the left hemisphere (indicated as a percentage).** Please note that lesions affecting the right hemisphere were flipped to the left for ease of comparison. The maximum overlap was observed within the internal capsule.

Twenty-five chronic stroke patients (20 male, 5 female, mean age = 66.68, std = 11.25; mean time post-stroke 33.05 months with a range from 10.57 to 81.57 months) formerly hospitalized at the University Hospital Cologne, Department of Neurology, 22 healthy age-matched controls (16 male, 6 female, mean age = 67.05, std = 6.59), and 24 healthy young control subjects recruited at the University of California, Santa Barbara (8 male, 16 female, mean age = 22.29, std = 3.66) were included in this study (see Supplementary Table 1 for patient information, Fig. 2 for a lesion overlay). For patients, inclusion criteria were as follows: (i) first-ever ischaemic stroke before 6 months or more; (ii) initial hand motor deficit; and (iii) age 40–90 years. Exclusion criteria were as follows: (i) any contraindications to MRI; (ii) cerebral haemorrhage; (iii) bihemispheric infarcts; (iv) reinfarction or any other neurological disease; as well as (v) persistence of severe aphasia or neglect. We further ensured that patients did not exhibit any pronounced orthopaedic conditions that could have prevented a proper assessment of residual motor impairment. All participants provided informed consent prior to participation. The local ethics committee approved the study carried out under the declaration of Helsinki.

### Motor tests

The Motricity Index (MI) was assessed in each patient to determine basal motor performance focusing on individual joints of the upper and lower limbs.^35^ For motor skills representing activities of daily living, we used the Action Research Arm Test (ARAT), including the subscales grasp, grip, pinch and gross movements.^36^ Thus, we covered a wide range of motor functions ranging from basal to complex motor control with widely used valid and reliable tests.^37-39^

### MRI acquisition

At both scanning sites, fMRI data were recorded using a Siemens MAGNETOM Prisma 3 Tesla scanner equipped with a 64-channel head coil (Siemens Medical Solutions, Erlangen, Germany). Of note, identical scanning protocols were used at both locations, rendering a potential bias introduced by varying scanning sites highly unlikely. DSI scans were sampled with a spatial resolution of 1.8*1.8*1.8 mm^3^ with b_max_-value of 5000 s/mm^2^, 128 diffusion directions and 10 additional b0s for *post hoc* movement correction (TR = 4300 ms, TE = 96 ms, FoV = 262 mm). In addition, T_1_-weighted images (TR = 2500 ms, TE = 2.22 ms, FoV = 241 mm, 208 axial slices, voxel size = 0.94*0.94*0.94 mm^3^) and T_2_-weighted images (TR = 3200 ms, TE = 0.566 ms, FoV = 241 mm, 208 axial slices, voxel size = 0.94*0.94*0.94 mm^3^) were acquired.

### MRI preprocessing

Preprocessing was performed using QSIPrep 0.13.0RC1,^40^ based on Nipype 1.6.0.^41^ The exact workflow is described in the printout from QSIPrep: The T_1_-weighted (T_1_w) image was corrected for intensity non-uniformity using N4BiasFieldCorrection as implemented in ANTs 2.3.1^42^ and used as T_1_w reference throughout the workflow. The T_1_w reference was then skull-stripped with antsBrainExtraction, using OASIS as a target template. Spatial normalization to the ICBM 152 Nonlinear Asymmetrical template version 2009c^43^ was performed through nonlinear registration with antsRegistration (ANTs 2.3.1),^44^ using brain-extracted versions of both T_1_w volume and template. Brain tissue segmentation of CSF, WM and grey matter was performed on the brain-extracted T_1_w using FAST as implemented in FSL.^45^

For diffusion data preprocessing, any images with a b-value <100 s/mm^2^ were treated as a *b* = 0 image. MP-PCA denoising as implemented in MRtrix3’s dwidenoise^46^ was applied with a 5-voxel window. B1 field inhomogeneity was corrected using dwibiascorrect from MRtrix3 with the N4 algorithm.^42^ After B1 bias correction, the mean intensity of the DWI series was adjusted so all the mean intensity of the *b* = 0 images matched across each separate DWI scanning sequence. Motion correction was performed using 3dSHORE as implemented in QSIPrep.^47^ The DWI time series were resampled to ACPC, generating a preprocessed DWI run in ACPC space with 1.8 mm isotropic voxels.

### DSI-reconstruction

Diffusion orientation distribution functions (ODFs) were reconstructed using generalized *q*-sampling imaging^48^ with a ratio of mean diffusion distance of 1.25. Next, individual gFA maps were created and normalized to the MNI standard space using ANTs.

### Lesion masking and whole-brain compartmentalization

**Figure 3:**
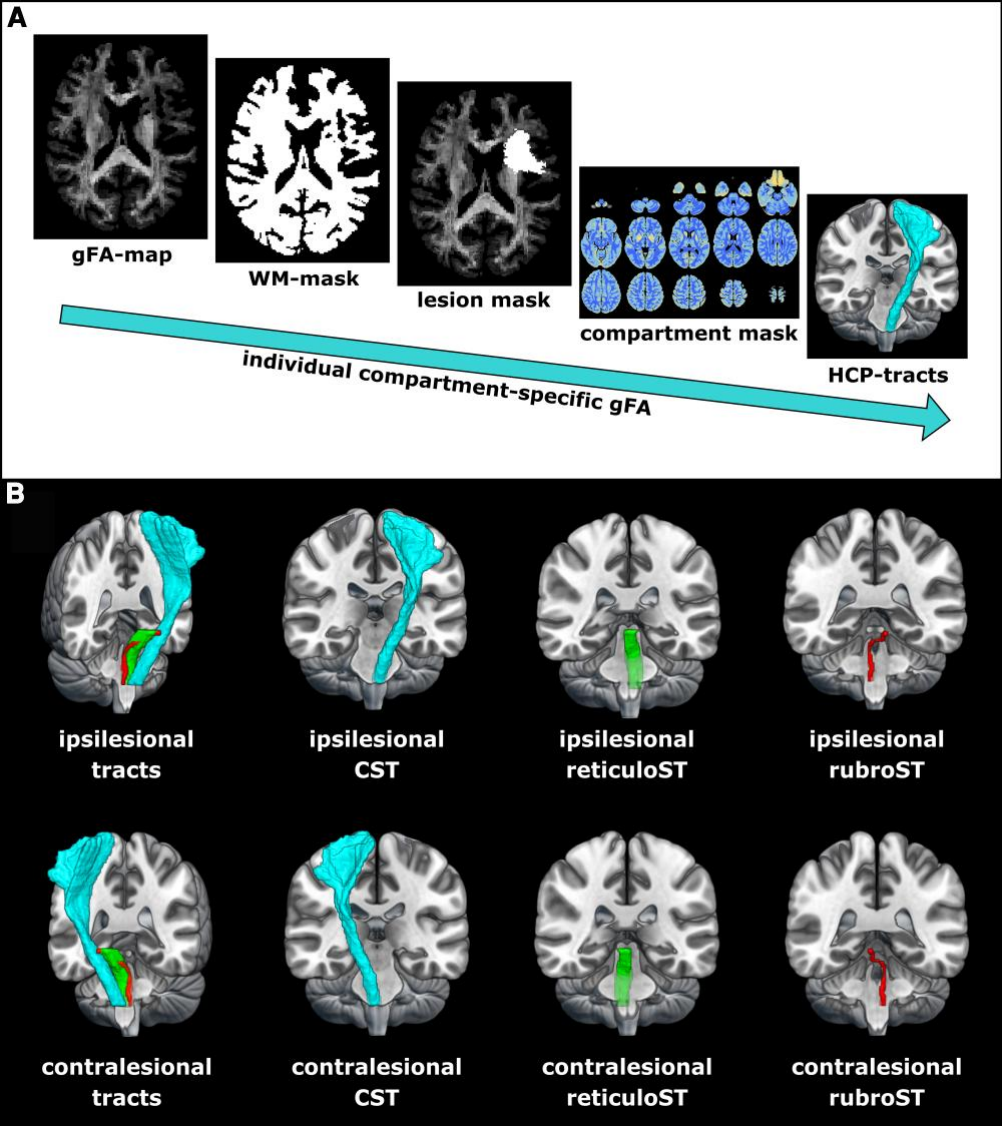
**Extraction of compartment-specific tractwise gFA.** (**A**) Workflow to obtain compartment-specific gFA values per tract. First, voxels containing WM were extracted from each subject’s normalized gFA-map using individual WM tissue classifications as derived from QSIPrep. For patient data, lesion masks were applied to focus the analysis on secondary degeneration processes rather than the assessment of anisotropy within the lesion. A deterministic compartment mask was applied to categorize all voxels according to the number of trackable directions.^10^ Mean gFA was extracted for descending motor tracts as defined by the HCP tractography atlas.^49^ (**B**) Motor tracts derived from the HCP tractography atlas^49^ used for gFA extraction. Note that ‘ipsilesional’ is defined by the origin of the tract superior to its decussation. Symmetric masks were used to ensure an equal number of voxels in each hemisphere, reducing potential sampling biases.

For patient data, lesion masks were drawn on individual T_2_-weighted images using MRIcron (www.sph.sc.edu/comd/rorden/Mricron) and applied to patients’ gFA-maps, thereby excluding direct lesion effects on gFA from further analyses to focus on secondary degeneration (cf. Fig. 3A). In case of right-hemispheric lesions, masks and gFA-maps were flipped along the mid-sagittal plane to ensure that all lesions were located in the left hemisphere, thereby rendering group comparisons possible.^50-52^ Moreover, individual WM-masks derived from QSIPrep were applied to each subject’s gFA-map, excluding non-WM voxels from further analyses. A deterministic mask denoting the number of trackable directions per voxel (constructed using *n* = 630 HCP data sets)^10^ was used to compartmentalize whole-brain gFA maps (cf. Fig. 1). Compartmentwise mean gFA values [(i) all voxels; voxels with (ii) one; (iii) two; and (iv) three fibre directions] were extracted from the motor tracts of interest (CST, rubroST, reticuloST) as defined in the HCP tractography atlas^49^ (cf. Fig. 3B, Supplementary Figure 1). To focus on descending fibres and exclude the widespread cortical inputs to the reticuloST, the reticuloST mask was trimmed, retaining only the part of the tract below *z* = −7. This ensured that reticuloST and rubroST masks commenced on the same *z*-level, ruling out systematic differences between the extrapyramidal masks. In order to avoid a sampling bias induced by different numbers of voxels in left- and right-hemispheric masks, we constructed symmetric masks by flipping left-hemispheric masks along the mid-sagittal plane and applied the resulting masks to the right hemisphere. A considerable overlap with other major fibre tracts such as the superior longitudinal fasciculus was excluded by visual inspection (cf. Supplementary Figure 2).

## Statistical analyses

### Differentiating stroke and ageing-related effects

To differentiate between ageing- and stroke-related effects, we compared the three subject groups across different compartments for the left and right CST. We computed a mixed ANOVA with the between factor group (levels: patients, age-matched controls, young controls) and the within factor compartment [levels: all (all voxels), one (one-directional voxels), two (two-directional voxels)] for the left and right CST. All assumptions for performing the ANOVA were met. A Greenhouse–Geisser correction was applied where appropriate. *Post hoc* pairwise *t*-tests were used to test for the following differences: (i) compartment all: patients versus old controls; (ii) compartment one: patients versus old controls; (iii) compartment two: patients versus old controls; (iv) compartment all: young versus old controls; (v) compartment one: young versus old controls; and (vi) compartment two: young versus old controls. Results of *post hoc* tests were FDR-corrected for the number of comparisons.

### Probing for extrapyramidal anisotropy differences

By comparing patients and age-matched controls with respect to mean gFA derived from extrapyramidal tracts, we probed for a potential reorganization of the extrapyramidal system. We computed a mixed ANOVA with the between factor group (levels: patients, age-matched controls) and the within factor compartment [levels: all (all voxels), one (one-directional voxels), two (two-directional voxels)]. A Greenhouse–Geisser correction was applied where appropriate.

### Explaining motor impairment: ipsilesional CST

In order to probe for a possible relationship with behaviour, we computed simple linear regressions with mean gFA derived from the ipsilesional CST as predictor and ARAT, MI arm or MI leg as the outcome variable. Next, we repeated these analyses using mean gFA derived from one- or two-directional voxels as predictors. Moreover, according analyses were performed using the compartment-specific asymmetry index as predictor, which was determined as asymmetry = [mean gFA(unaff CST)—mean gFA(aff CST)]/[mean gFA(unaff CST) + mean gFA(aff CST)].^53^ This step was implemented to improve the signal-to-noise ratio by accounting for the individual ageing-related level of atrophy.^16,53^ To compare our current results with more conventional ROI-based approaches, we computed regression models using gFA derived from (i) all voxels contained in the PLIC^17-19^ and (ii) voxels that fell into the CST section from the mesencephalon to the CPs (*z* from −25 to −20).^54^ As previous research has shown that the microstructural integrity of the CST and motor impairment exhibit a stronger relationship in patients with persisting motor deficits,^4,55^ we repeated the compartmentwise analyses for a non-fully recovered subgroup as defined by an ARAT score < 57.^36^ For each step, all *P*-values were FDR-corrected for the number of comparisons.

### Explaining motor impairment: contralesional CST and extrapyramidal pathways

The role of alternative motor pathways in motor function after stroke was assessed through linear regression analyses. To test for a relationship with basal motor performance, five linear regression models were computed with the MI arm score as the outcome variable and contralesional CST, ipsilesional reticuloST, contralesional reticuloST, ipsilesional rubroST or contralesional rubroST as the predictor variable. The resulting *P*-values were FDR-corrected for the number of comparisons. These analyses were carried out for mean gFA derived from (i) all voxels; (ii) one-directional voxels; and (iii) two-directional voxels. The same procedure was repeated for the ARAT score and the MI leg score as outcome variable to probe for a potential relationship with the performance of activities of daily living or lower limb performance, respectively. Next, we tested for shared variance between alternative motor pathways and the ipsilesional CST by entering mean gFA values derived from extrapyramidal tracts that correlated with motor performance as predictor variables into the regression model containing gFA derived from one-directional ipsilesional CST voxels. To rule out multicollinearity of predictor variables, we probed for a correlation between extrapyramidal anisotropy of two-directional voxels and CST anisotropy of one-directional voxels. An overlay of all four extrapyramidal tracts was created to test whether compartment two of the four tracts captured a high number of identical voxels.

## Results

### Ageing- versus stroke-related CST anisotropy

**Figure 4:**
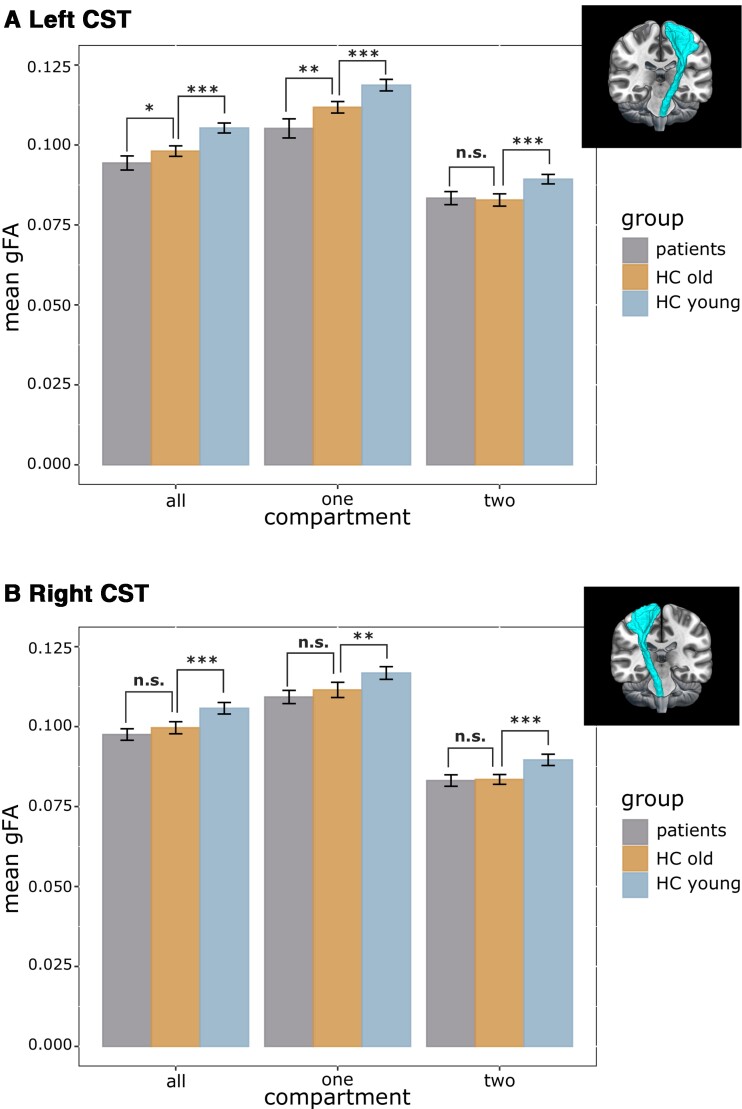
**Group differences per compartment.** Results of a mixed ANOVA with the between factor group (levels: patients, age-matched controls, young controls) and the within factor compartment [levels: all (all voxels), one (one-directional voxels), two (two-directional voxels)] for (**A**) the ipsilesional CST and (**B**) the contralesional CST. (**A**) Left (ipsilesional) CST: While young and old controls differed across all compartments, patients and age-matched controls differed for compartment 1 but not for compartment 2. Thus, ageing-related changes occurred across the entire left CST entailing both one- and two-directional voxels, while stroke-related decreases in gFA were driven by one-directional voxels. (**B**) Right (contralesional) CST: In line with findings for the left CST, young and old controls differed across all compartments, yet there was no difference between patients and age-matched controls. *Post hoc* two-sided *t*-tests were used to further investigate significant effects. Significance thresholds: *** *P* < 0.001, ** *P* < 0.01, * *P* < 0.05 (FDR-corrected for multiple comparisons). Error bars represent two standard errors.

For the ipsilesional CST, we found a significant main effect of group (F(2, 68) = 32.28, *P* < 0.001, generalized η^2^ = 0.44) and compartment (F(1.05, 71.56) = 2178.42, *P* < 0.001, generalized η^2^ = 0.84), as well as an interaction between group and compartment (F(2.10, 71.56) = 19.77, *P* < 0.001, generalized η^2^ = 0.09). *Post hoc* independent sample *t*-tests showed that patients featured reduced gFA values within the ipsilesional CST compared with age-matched controls when considering all voxels within the CST mask (t(45) = −2.65, *P* = 0.013, FDR-corrected). This difference was attributable to voxels containing only one fibre direction (t(45) = -3.66, *P* = 0.001, FDR-corrected), while two-directional voxels showed no difference (t(45) = 0.41, *P* = 0.684, FDR-corrected; cf. Fig. 4A). At the same time, young and old controls differed with respect to all levels of the factor compartment (all *P* < 0.001, FDR-corrected), indicating that the age-related difference could be objectified in all compartments, affecting both one- and two-directional voxels. For the contralesional CST, we found a main effect of group (F(2, 68) = 19.24, *P* < 0.001, generalized η^2^ = 0.34) and a main effect of compartment (F(1.06, 71.80) = 3756.75, *P* < 0.001, generalized η^2^ = 0.86). There was no interaction between group and compartment (F(2.11, 71.80) = 2.41, *P* = 0.094). While patients and age-matched controls did not differ with respect to mean gFA in any compartment (*P* > 0.1), young and elderly control subjects differed across all compartments (*P* < 0.01, FDR-corrected; cf. Fig. 4B). Thus, while stroke-related changes in anisotropy were specific to the ipsilesional CST, ageing-related degeneration affected the CST in both hemispheres.

### Anisotropy in the extrapyramidal system

For the extrapyramidal system entailing bihemispheric reticuloST and rubroST, there was a main effect of compartment (F(1.01, 45.29) = 656.22, *P* < 0.001, generalized η^2^ = 0.38) but no main effect of group (F(1,45) = 0.01, *P* = 0.913) or interaction between group and compartment (F(1.01,45.29) = 0.78, *P* = 0.381), indicating that patients and age-matched controls did not differ with respect to extrapyramidal anisotropy.

### Ipsilesional CST degeneration explains motor impairment

**Figure 5:**
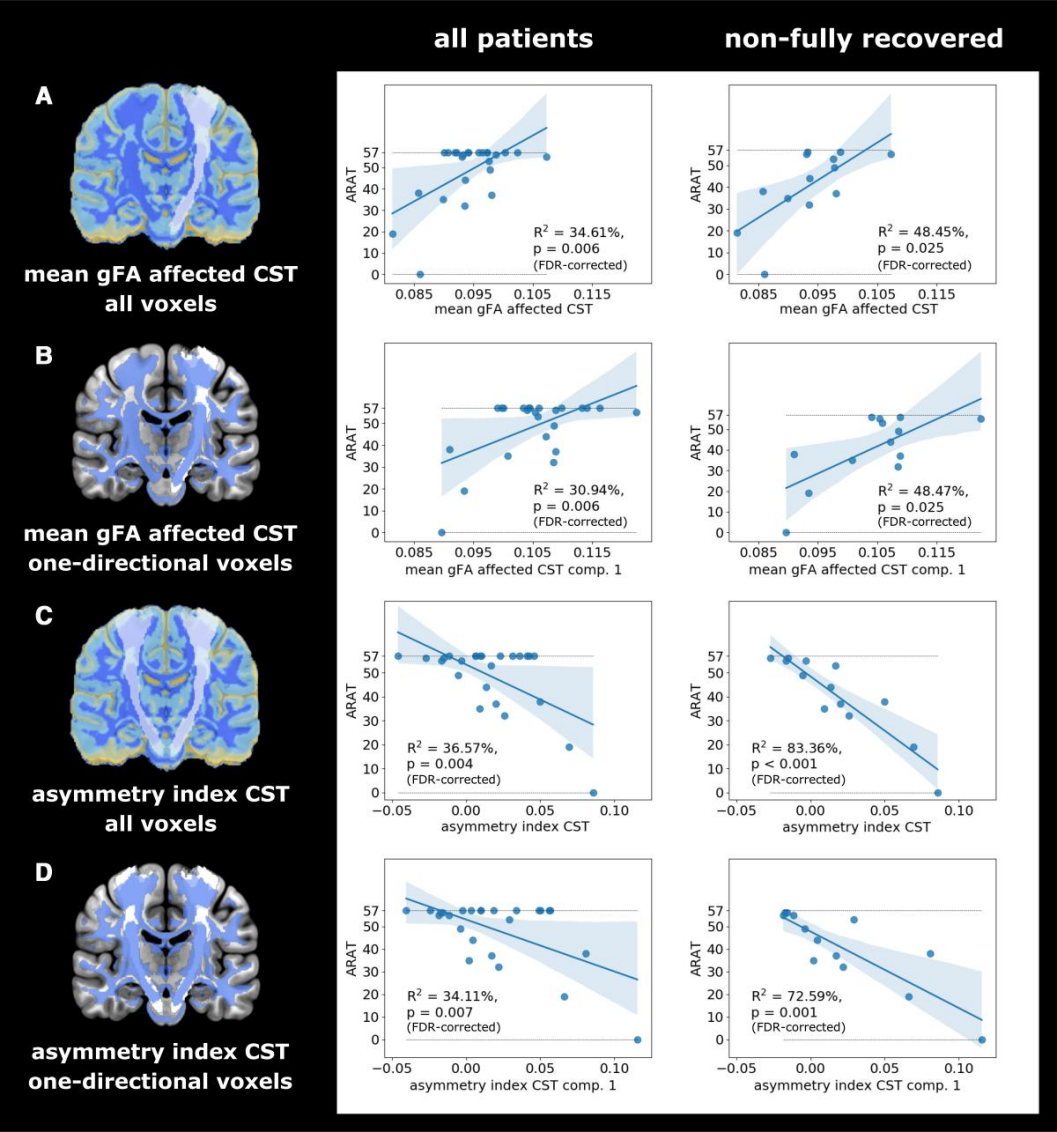
**Regression analyses for the association between different gFA-based CST measures and ARAT motor scores**. (**A**) Mean gFA values within the ipsilesional CST explained motor impairment for the entire cohort and to an even higher degree for the non-fully recovered subgroup. (**B**) By obtaining the mean gFA value from one-directional voxels within the ipsilesional CST, it becomes evident that the association between anisotropy and motor performance was driven by descending fibres. Note that the mean taken from two-directional voxels did not significantly explain motor performance (*P* > 0.2, FDR-corrected). (**C**) To improve the signal-to-noise ratio, we calculated the asymmetry index that accounts for the subject-specific level of atrophy by considering both the affected and unaffected CST. Across all CST voxels, we observed a significant association with the ARAT score for the entire sample and the non-fully recovered subgroup. (**D**) Entering the asymmetry index from one-directional voxels as the predictor into the regression models yielded a similar ratio of explained variance as in (**C**).

Mean gFA values derived from all voxels of the ipsilesional CST significantly explained upper limb impairment as measured by the ARAT (R^2^ = 34.61, *P* = 0.006, FDR-corrected) or MI arm (R^2^ = 28.9%, *P* = 0.008, FDR-corrected) (cf. Fig. 5). The regression model containing MI leg as the outcome variable showed a trend towards significance (R^2^ = 15.25%, *P* = 0.054, FDR-corrected). Repeating the analyses with only one-directional voxels showed that the results were indeed driven by descending fibres (ARAT: R^2^ = 30.94%, *P* = 0.006; MI arm: R^2^ = 31.94%, *P* = 0.010; MI leg: R^2^ = 17.71%, *P* = 0.036, FDR-corrected). Regression models using mean gFA derived from compartment two did not reach significance (all *P* > 0.2, FDR-corrected; for a summary of all results see Supplementary Table 3). Using the asymmetry index based on the entire CST as the predictor yielded a slightly higher percentage of explained variance for the ARAT (R^2^ = 36.57%, *P* = 0.004, FDR-corrected) but not for MI arm (R^2^ = 28.10%, *P* = 0.010, FDR-corrected) or MI leg (R^2^ = 11.21%, *P* = 0.102, FDR-corrected). Increased explained variance was also observed when computing the asymmetry index for one-directional voxels (ARAT: R^2^ = 34.11%, *P* = 0.007; MI arm: R^2^ = 31.78%, *P* = 0.005; MI leg: R^2^ = 16.01%, *P* = 0.048, FDR-corrected). Of note, using gFA derived from one-directional voxels outperformed conventional ROI-based approaches which rely on anisotropy from voxels regardless of their directional compartment (for detailed results see Supplementary Table 2).

When exclusively considering patients with persisting motor deficits and repeating the analyses for this non-fully recovered subgroup, the ratio of explained variance increased for all models, when using mean gFA across all voxels (ARAT: R^2^ = 48.45%, *P* = 0.025; MI arm: R^2^ = 40.77%, *P* = 0.028; MI leg: R^2^ = 29.12%, *P* = 0.057, FDR-corrected) or gFA extracted from one-directional voxels (ARAT: R^2^ = 48.47%, *P* = 0.025; MI arm: R^2^ = 46.22%, *P* = 0.016; MI leg: R^2^ = 35.11%, *P* = 0.033, FDR-corrected), as well as the asymmetry index derived from all voxels (ARAT: R^2^ = 83.36%, *P* < 0.001; MI arm: R^2^ = 63.08%, *P* = 0.002; MI leg: R^2^ = 50.88%, *P* = 0.006, FDR-corrected) or one-directional voxels (ARAT: R^2^ = 72.59%, *P* = 0.001; MI arm: R^2^ = 63.08%, *P* = 0.002; MI leg: R^2^ = 52.47%, *P* = 0.005, FDR-corrected).

### Extrapyramidal pathways explain motor impairment

**Figure 6:**
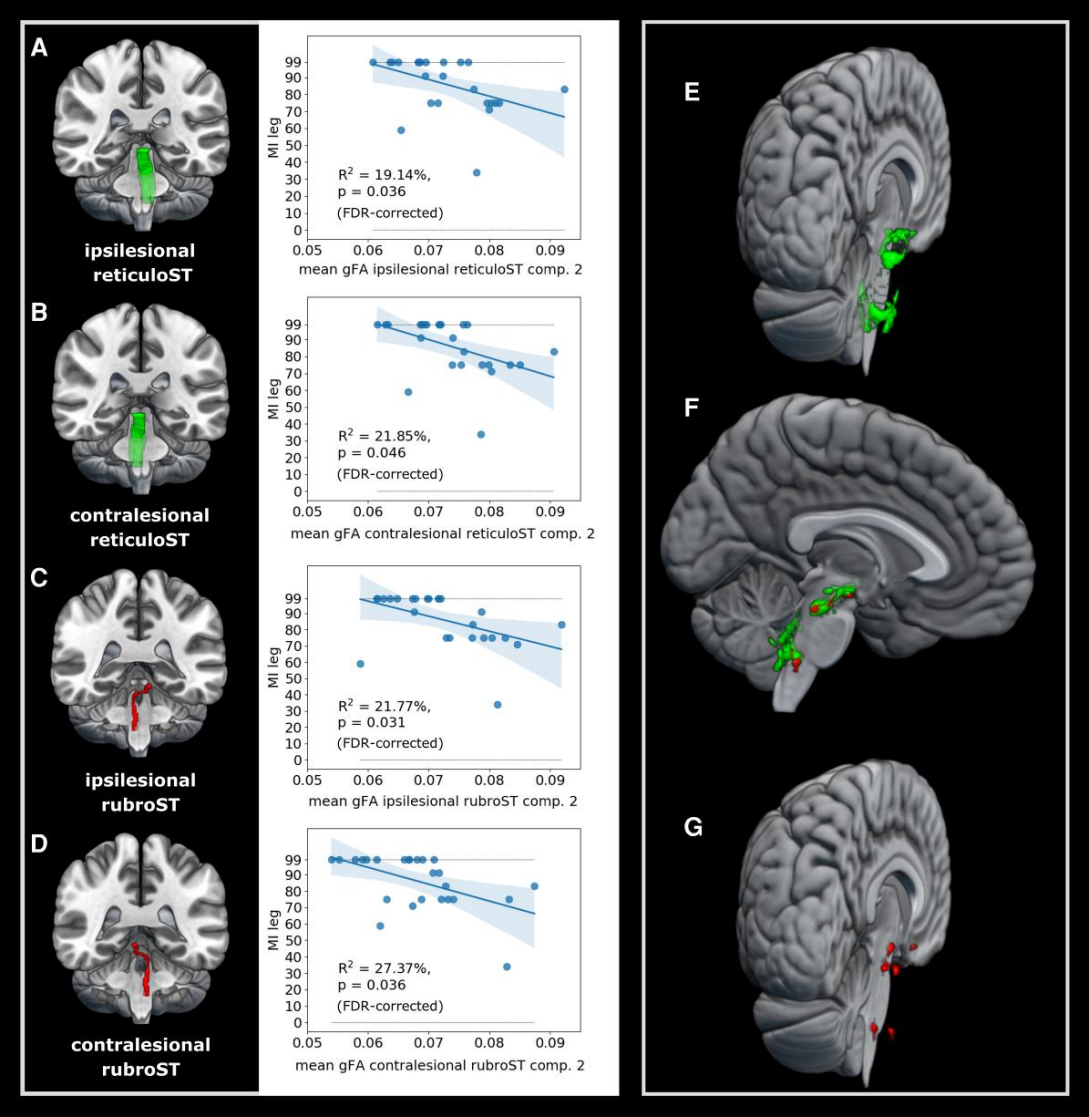
**Explained variance by mean gFA derived from two-directional voxels of extrapyramidal pathways for the motricity index lower extremity scores.** Note that there was a robust negative relationship between leg impairment and gFA derived from two-directional voxels within the (**A**) ipsilesional reticuloST, (**B**) contralesional reticuloST, (**C**) ipsilesional rubroST, and (**D**) contralesional rubroST. Of note, compartment 2 voxels of the reticuloST (**E**) and rubroST (**G**) did not contain many overlapping voxels (**F**).

While neither the contralesional CST nor any of the extrapyramidal tracts explained variance in ARAT scores in any compartment (*P* > 0.7, FDR-corrected), the rubroST descending from the contralesional hemisphere showed a significant relationship with the MI arm score for two-directional voxels (R^2^ = 25.94%, *P* = 0.047, FDR-corrected) and a trend towards significance when using all voxels (R^2^ = 22.18, *P* = 0.087, FDR-corrected). For the MI leg score, all extrapyramidal tracts explained variance in motor performance when using two-directional voxels (all *P* < 0.05), but not the contralesional CST (cf. Table 1, Supplementary Table 3, and Fig. 6A-D). Notably, the two-directional voxels of the four extrapyramidal tracts barely overlapped, with only seven voxels being included in all four masks, indicating that the explained variance observed for the different tracts was not driven by the same set of overlapping voxels (cf. Fig. 6E-G).

**Table 1:** Linear regression results of the motricity index leg score for alternative motor output pathways using mean gFA derived from two-directional voxels

Predictor	R^2^	*P* (FDR)
Contralesional CST	1.11%	0.616
Ipsilesional reticuloST	19.14%	0.036
Contralesional reticuloST	21.85%	0.046
Ipsilesional rubroST	21.77%	0.031
Contralesional rubroST	27.37%	0.036

To assess whether the extrapyramidal system’s anisotropy may reflect a compensatory mechanism, i.e. may hold additional information on motor outcome exceeding that of the ipsilesional CST, we tested whether the extrapyramidal system explained additional behavioural variance when combined with the ipsilesional CST. Adding mean gFA derived from compartment 2 of any of the extrapyramidal tracts as an additional variable to anisotropy derived from one-directional ipsilesional CST voxels into a regression model significantly increased the amount of explained variance for the MI leg score, underlining the independence of extrapyramidal tracts from the ipsilesional CST (all R^2^ values > 38%, *P* < 0.01, cf. Supplementary Table 4). Concerning the shared variance between ipsilesional CST and extrapyramidal system in the prediction of gross upper limb performance, the combination of mean gFA derived from compartment 1 of the ipsilesional CST and compartment 2 of the contralesional rubroST led to a significantly higher ratio of explained variance than any of the two tracts alone (R^2^ = 51.02%, *P* < 0.001). Of note, individual predictor variables were not correlated, ruling out biases due to multicollinearity (cf. Supplementary Figure 3).

## Discussion

Using DSI data combined with a compartmentwise analysis approach that differentiates the number of fibre directions per voxel, we found that decreased anisotropy following stroke primarily affected one-directional voxels of the ipsilesional CST while ageing-related degeneration was observed across all directional compartments. In line with numerous previous findings, ipsilesional CST anisotropy explained motor performance across many functional domains, which entirely relied on one-directional voxels representing descending fibres. Thus, our data provide direct evidence for Wallerian degeneration occurring throughout the entire ipsilesional CST and underline the seminal pathophysiological role of Wallerian degeneration for various aspects of motor function. However, the ipsilesional CST cannot be considered the sole descending motor pathway involved in motor control post-stroke: anisotropy of extrapyramidal tracts was associated with specific aspects of motor impairment, highlighting function-specific compensatory roles of distinct pathways. While the contralesional rubroST was indicative of gross motor control of the arm, bihemispheric rubroST and reticuloST were related to lower limb motor function. Importantly, the relationship between extrapyramidal tracts and motor performance only emerged when focusing on voxels containing two fibre directions, which may explain contradictory findings of previous studies. Of note, stroke patients did not differ from age-matched controls regarding anisotropy in extrapyramidal tracts, suggesting that functional compensation through extrapyramidal pathways does not rely on reorganization of these tracts but rather reflects an aspect of the structural reserve of the motor system as discussed below.

### Structural CST alterations in stroke and ageing

Using a compartmentwise approach, we aimed to disentangle the drivers of anisotropy changes in chronic stroke patients to further elucidate the potential of anisotropy in descending motor tracts to explain motor control after stroke. In line with our hypotheses, the expected decline in anisotropy in the ipsilesional CST compared with age-matched controls was limited to voxels containing one-directional, i.e. descending fibres, and could not be observed in the contralesional CST (cf. Fig. 4). Conversely, age-related differences between the young and old control group manifested themselves in bilateral CSTs regardless of the number of intravoxel directions, which nicely fits the notion that ageing represents a global phenomenon affecting more than just descending fibre tracts.^20^ Thus, the compartmentwise analysis approach allowed us to differentiate ageing- and stroke-related anisotropy changes. Of note, decreased ipsilesional CST anisotropy in stroke patients compared with age-matched controls was only evident in one-directional voxels, highlighting Wallerian degeneration as the main driver of anisotropy changes in descending ipsilesional CST fibres. The pivotal influence of Wallerian degeneration on anisotropy changes post-stroke clarifies why previous research has frequently linked the integrity of ipsilesional CST fibres to motor impairment. For instance, Schaechter *et al.*^23^ investigated differences between chronic stroke patients and healthy controls by comparing FA curves extracted along the ipsilesional CST starting from the precentral gyrus down to the CP. Their results indicate that poorly recovered stroke patients feature significantly decreased FA values in the ipsilesional CST between the height of the PLIC and CP. Parts of this CST section have repeatedly been used as ROIs to investigate stroke-related WM abnormalities and their association with motor impairment. As Pierpaoli *et al.*^11^ point out, this particular stretch of the CST is characterized by densely packed descending fibres without any significant association tracts passing through. In other words, it mainly consists of descending fibres which we captured as one-directional voxels in our analyses.

Interestingly, our results suggest that focusing on descending fibres along the entire CST might increase the sensitivity for certain aspects of motor performance: When using all CST voxels, mean gFA significantly explained motor impairment of the upper but not the lower limb. However, lower limb deficits could also be explained when exclusively including one-directional voxels. Moreover, the significant association between gFA in the ipsilesional CST and motor impairment of the upper limb was entirely driven by one-directional voxels: While mean gFA derived from one-directional voxels accounted for almost the same amount of explained variance as all CST voxels combined, two-directional voxels did not show any association with motor impairment. Of note, these effects were intensified for the non-fully recovered subgroup, which is in line with previous studies reporting more severe WM changes and better predictions of motor performance in patients with more pronounced motor deficits.^4,55^ The ratio of explained variance was even higher when using the asymmetry index and thereby accounting for a patient’s individual level of ageing-related atrophy.

Importantly, the present findings do not only underline the applicability of a DSI-based compartmentwise approach but also offer a possible solution to the problem of arbitrary ROI selection since focusing on descending fibres allowed us to include the entire length of the CST into our analysis. Moreover, using one-directional voxels from the entire length of the ipsilesional CST may also help to reduce ageing-related confounds commonly introduced when focusing on regions heavily affected by ageing-related atrophy such as the PLIC.^21,22^ In summary, anisotropy of one-directional ipsilesional CST fibres primarily reflects Wallerian degeneration of descending motor fibres which accounted for a large amount of variance in motor impairment across various domains of motor control. However, our current results also highlight that Wallerian degeneration of the ipsilesional CST should not be regarded as the only factor contributing to motor control after stroke, given the compensatory potential of other descending motor pathways.

### Compensatory role of the extrapyramidal system

The role of the extrapyramidal system in motor recovery following stroke is a subject of ongoing debate, inspired by several studies reporting associations between motor impairment and extrapyramidal tract anisotropy. While some authors argue that increased reliance on the extrapyramidal system may help patients to recover successfully,^4,29^ others interpret their findings as maladaptive reorganization.^24,27,28,56^ These opposing interpretations are largely driven by the direction of the observed correlations between anisotropy and motor behaviour: positive correlations linking higher anisotropy to better motor performance are often construed as beneficial compensation, whereas negative correlations linking higher anisotropy to worse motor performance are commonly interpreted as maladaptive overcompensation caused by reorganization processes of the extrapyramidal tracts. A commonly held view is that the overcompensation may stem from an overactivation of extrapyramidal pathways resulting in increased flexor synergies, hindering the control of individual movements.^24,25^ Here, we exclusively observed negative associations between extrapyramidal anisotropy and motor performance of the arm and leg (cf. Fig. 6). Therefore, one may interpret our current findings as a maladaptive overcompensation by the extrapyramidal system. However, motor impairment across patients was exclusively explained by two-directional voxels which renders the interpretation more difficult. Two-directional voxels are characterized by two dominant directions which need to change to a different extent for gFA to either de- or increase. Depending on the ratio of the two directions, even an underlying decrease in one direction could lead to an overall increase in gFA. For instance, if only the non-dominant direction in a two-directional voxel decreases while the dominant direction remains constant, the gFA value will increase. Following this logic, gFA values will not change at all when both fibre directions within a two-directional voxel change to the same extent. Thus, a higher or lower degree of anisotropy in two-directional voxels should not be mistaken for higher or lower microstructural integrity. In other words, higher or lower degrees of anisotropy in two-directional voxels cannot be functionally interpreted in a straightforward way, which may well account for previous contradictory findings. Therefore, future studies should focus on changes at the subvoxel level to further our understanding of the underlying pathophysiological mechanisms.

From a functional perspective, extrapyramidal pathways are thought to support gross motor function via their projections to proximal muscles of the arm and leg. In particular, the basal motor control by extrapyramidal tracts may stem from their ability to directly code for strength of muscle activation as recently observed for the reticular formation in monkeys.^57^ Our current results are well in line with this notion, as reflected by their associations with MI scores of the upper and lower limb but not for the ARAT. For the ARAT, higher scores can be achieved through compensatory strategies applied in daily life, which requires higher degrees of motor control and more complex motor skills.^58^ The MI, on the other hand, assesses each joint individually. In other words, it relies on muscular strength and therefore reflects more basal demands on the motor system.^59,60^ Given the observed relationship between anisotropy of all four extrapyramidal tracts and the patients’ ability to move individual joints as assessed using the MI, our current findings underline the potential involvement of extrapyramidal pathways in the recovery of gross motor function after stroke. This notion is supported by the fact that extrapyramidal pathways seemed to be independent of the ipsilesional CST as indicated by the increase in explained variance when combining CST and extrapyramidal anisotropy in a regression model explaining MI scores. These observations perfectly match previous reports indicating an additional explanation of variance in motor function by anisotropy of the reticuloST and rubroST independent of the ipsilesional CST.^29,31^ Thus, these findings are in line with the notion that both pyramidal and extrapyramidal tracts contribute independently to the execution and control of gross motor function. To further elucidate the mechanistical role of extrapyramidal tracts in motor control post-stroke, a seminal question lies in whether extrapyramidal tracts undergo stroke-induced reorganization or whether compensation may be determined by the premorbid level of descending motor output. Considering that we did not find a group difference between stroke patients and age-matched controls, a compensatory upregulation through structural changes of the extrapyramidal tracts seems less likely. Conversely, extrapyramidal anisotropy may rather indicate a structural reserve within those tracts the motor system may capitalize on to compensate for stroke-induced damage.^32^

### Anatomical foundations of extrapyramidal compensation

Having established a link between motor function and extrapyramidal integrity of two-directional voxels, the question arises which anatomical parts of the reticuloST and rubroST drove this observation. The reticuloST receives its cortical inputs from the primary motor cortex, as well as the premotor and supplementary motor areas^61^ and descends mostly ipsilaterally from the medial pontine and medullary reticular formation.^62^ The rubroST originates from the red nucleus at the level of the mesencephalon and receives inputs from an array of cortical areas and the cerebellum. It decussates at the level of the red nucleus and descends alongside the lateral CST.^63^ While most fibres within the rubroST decussate, most of the reticuloST descends without crossing. Therefore, it seems striking that tracts from both hemispheres showed a similar relationship with motor performance. A potential explanation for this observation might derive from the limited number of crossing fibres in both tracts.^5,64^ Considering the anatomical proximity of these crossing fibres and the relatively high overlap in explained variance in motor performance of all four tracts, one might assume that identical voxels were included in both tracts due to tracking inaccuracies or sampling biases. However, computing the overlap between all four masks yielded a negligible number of shared voxels.

When visualizing compartment two voxels (cf. Fig. 6E-G), clusters emerged at three different levels. Voxels located at the most superior level may potentially constitute input and output fibres from various nuclei. For example, anisotropy surrounding the red nucleus has been shown to correlate with motor impairment,^4,26,28^ supporting the notion that output properties of the red nucleus may contribute to motor control after stroke. Moreover, units in the pontine reticular formation of the cat discharge during motor activity even when deprived of any other stimulus inputs,^65^ which highlights the involvement of the pontine reticular formation and the descending reticuloST for the generation of motor output. Second, various clusters can be seen close to the midline, where both rubroST and the reticuloST cross over to the other hemisphere. Whether the integrity of these crossings is vital for motor performance after stroke remains an interesting question for future research. Third, at the lowest level, a cluster of two-directional voxels may include fibres from cerebellar structures. Cortico-cerebellar pathways as well as the cerebellar peduncles have already been shown to relate to residual motor function after stroke,^66-68^ and it may thus be possible that the observed relationship with motor impairment was partially driven by fibres projecting to or from the cerebellum. In summary, our methodological approach helped to identify specific parts of the extrapyramidal system contributing to compensation of gross arm and leg movements after stroke, primarily comprising fibres crossing the midline, fibres potentially mitigating output from brain stem nuclei (such as the red nucleus) or interactions across different parts of the extrapyramidal system and cerebellum.

### Limitations and future directions

One major limitation pertains to the limited sample size. However, we covered a wide range of motor deficits and lesion sizes and a sample of 25 stroke patients is well in the range of other hypothesis-driven fMRI studies.^30,50,52^ Thus, while a bigger sample would be desirable, our sample yields sufficient variability for the present analysis framework, resulting in meaningful effects of considerable sizes. Moreover, the focus on chronic stroke patients allowed us to draw conclusions regarding the effects of secondary degenerative processes and to assess the reorganized motor system. Further studies are warranted to quantify anisotropy changes in descending tracts longitudinally starting in the acute phase after stroke. As our findings suggest that compensatory processes in the extrapyramidal system rely on a patient’s structural reserve rather than structural changes within the extrapyramidal tracts, estimating the propensity of these tracts to compensate for lost functionality based on anisotropy measures might already be possible in the acute phase post-stroke. Moreover, the present study focused on structural alterations in descending tracts underlining their relationship with motor impairment. Given that compensatory mechanisms may also operate via alternative routes such as cortico-(sub)cortical interactions, extending the current analyses by assessing structural connectivity between different (sub)cortical areas may result in a more comprehensive picture.^69^ Since motor impairment has also been shown to be closely related to functional connectivity between cortical motor regions,^50,70-74^ future research should try to elucidate the relationship between stroke-related changes in the structural and functional organization of the motor network.

## Conclusion

By disentangling ageing from stroke-related effects via compartmentwise analyses, we provide direct evidence for Wallerian degeneration of the ipsilesional descending CST and its seminal role in various aspects of motor control of the upper and lower limbs. Anisotropy of the contralesional rubroST explained gross motor performance of the affected hand, while anisotropy within all extrapyramidal tracts located throughout the brainstem was linked to motor function of the lower limb in chronic stroke patients, supporting the notion of increased reliance on extrapyramidal pathways to support basal motor function after CST damage. Of note, all extrapyramidal tract findings were based on two-directional voxels which can be found in specific anatomical locations throughout the brainstem, potentially mitigating output of brainstem nuclei, signals crossing the midline and cerebellar influences. Since the highest ratio of explained variance was achieved when combining extrapyramidal pathways and ipsilesional CST anisotropy, clinical biomarkers should consider both the degeneration of the ipsilesional CST, as well as the compensation via the extrapyramidal system. From a mechanistic perspective, our findings suggest that compensatory processes in the extrapyramidal system reflect an aspect of structural motor reserve rather than reorganization of extrapyramidal tracts. In summary, anisotropy of descending motor pathways seems to be a promising marker for motor impairment post-stroke, especially when divided into compartments based on the number of trackable directions per voxel.

## Supplementary Material

fcac301_Supplementary_Data

## Data Availability

Data are available from the corresponding author upon reasonable request.

## Abbreviations

CP =: cerebral peduncle
CST =: corticospinal tract
dMRI =: diffusion magnetic resonance imaging
DSI =: diffusion spectrum imaging
DTI =: diffusion tensor imaging
FA =: fractional anisotropy
gFA =: generalized fractional anisotropy
ODF =: orientation distribution function
PLIC =: posterior limb of the internal capsule
reticuloST =: reticulospinal tract
ROI =: region of interest
rubroST =: rubrospinal tract
WM =: white matter
