# Supplementary material for: The role of corticospinal and extrapyramidal pathways in motor impairment after stroke
Source: Brain Commun. 2022 Nov 21;5(1):fcac301. doi: 10.1093/braincomms/fcac301 (PMC9798285; doi:10.1093/braincomms/fcac301)
Supplement: fcac301_Supplementary_Data [file fcac301_supplementary_data.pdf]

Supplement

Supplementary Tables

Supplementary Table 1: Patient demographics. Our patient cohort comprised 12 fully recovered and 13 non-fully recovered patients as determined by the ARAT-score (fully recovered = 57 points, non-fully recovered < 57 points).

subject	*sex*	*affected hemisphere*	*ARAT*	*MI-arm*	*MI-leg*
1	m	l	19	65	59
2	f	r	57	83	83
3	m	r	38	76	75
4	f	r	56	91	75
5	m	l	57	99	91
6	m	l	35	92	99
7	f	l	32	77	83
8	f	r	57	76	75
9	m	r	49	91	99
10	f	l	56	76	75
11	m	r	57	91	99
12	m	l	57	99	99
13	m	l	55	92	91
14	m	r	57	99	99
15	m	r	57	99	99
16	m	r	53	99	99
17	m	l	55	92	99
18	m	l	44	83	75
19	m	r	37	84	75
20	m	l	57	99	99
21	m	l	57	99	99
22	m	l	0	34	34
23	m	r	57	99	71
24	m	l	57	99	99
25	m	r	57	99	99

Supplementary Table 2: Comparison of compartmentwise and ROI-based approaches. The first column depicts results when using one-directional voxels from the entire length of the CST. This is contrasted by two conventional ROI-based approaches using either the section from the mesencephalon to the cerebral peduncle (CP; z-level -25 to -20) or the posterior limb of the internal capsule (PLIC; z-level -5 to 20).

**one-directional CST voxels**			**mesencephalon to CP**			**PLIC**		
**DV**	**R2**	**p(FDR)**	**DV**	**R2**	**p(FDR)**	**DV**	**R2**	**p(FDR)**
**ARAT**	**0.309**	**0.006**	ARAT	0.081	0.500	ARAT	**0.339**	**0.007**
**MI-arm**	**0.319**	**0.010**	MI-arm	0.017	0.800	MI-arm	**0.187**	**0.046**
**MI-leg**	**0.177**	**0.036**	MI-leg	0.003	0.789	MI-leg	0.044	0.313

Supplementary Table 3: Overview of regression results. Behavioral variance in motor impairment is explained by compartment-wise anisotropy from descending motor tracts. Bold font indicates significance after FDR-correction.

all voxels				one-directional voxels				two-directional voxels			
DV	predictor	R2	p (FDR)	DV	predictor	R2	p (FDR)	DV	predictor	R2	p (FDR)
**ARAT**	**il CST**	**0.346**	**0.006**	**ARAT**	**il CST**	**0.309**	**0.006**	ARAT	il CST	0.126	0.243
**MI-arm**	**il CST**	**0.289**	**0.008**	**MI-arm**	**il CST**	**0.319**	**0.010**	MI-arm	il CST	0.059	0.364
MI-leg	il CST	0.152	0.054	**MI-leg**	**il CST**	**0.177**	**0.036**	MI-leg	il CST	0.010	0.638
**ARAT**	**CST asym**	**0.366**	**0.004**	**ARAT**	**CST asym**	**0.341**	**0.007**	ARAT	CST asym	0.141	0.193
**MI-arm**	**CST asym**	**0.281**	**0.010**	**MI-arm**	**CST asym**	**0.318**	**0.005**	MI-arm	CST asym	0.056	0.380
MI-leg	CST asym	0.112	0.102	**MI-leg**	**CST asym**	**0.160**	**0.048**	MI-leg	CST asym	0.000	0.925
ARAT	cl CST	0.000	0.956	ARAT	cl CST	0.001	0.892	ARAT	cl CST	0.006	0.890
ARAT	il RST	0.004	1.293	ARAT	il RST	0.002	1.054	ARAT	il RST	0.011	1.533
ARAT	cl RST	0.000	1.188	ARAT	cl RST	0.003	1.307	ARAT	cl RST	0.007	1.147
ARAT	cl rubroST	0.086	0.776	ARAT	cl rubroST	0.074	0.941	ARAT	cl rubroST	0.091	0.719
ARAT	il rubroST	0.005	1.852	ARAT	il rubroST	0.012	1.522	ARAT	il rubroST	0.001	0.871
MI-arm	cl CST	0.000	0.939	MI-arm	cl CST	0.000	0.936	MI-arm	cl CST	0.004	0.752
MI-arm	il RST	0.083	0.407	MI-arm	il RST	0.071	0.494	MI-arm	il RST	0.090	0.240
MI-arm	cl RST	0.055	0.432	MI-arm	cl RST	0.033	0.638	MI-arm	cl RST	0.124	0.211
MI-arm	cl rubroST	0.222	0.087	MI-arm	cl rubroST	0.184	0.161	**MI-arm**	**cl rubroST**	**0.259**	**0.047**
MI-arm	il rubroST	0.029	0.522	MI-arm	il rubroST	0.014	0.709	MI-arm	il rubroST	0.071	0.248
MI-leg	cl CST	0.008	0.676	MI-leg	cl CST	0.003	0.792	MI-leg	cl CST	0.011	0.616
MI-leg	il RST	0.133	0.121	MI-leg	il RST	0.100	0.205	**MI-leg**	**il RST**	**0.191**	**0.036**
MI-leg	cl RST	0.148	0.144	MI-leg	cl RST	0.123	0.215	**MI-leg**	**cl RST**	**0.218**	**0.046**
MI-leg	cl rubroST	0.176	0.184	MI-leg	cl rubroST	0.130	0.384	**MI-leg**	**cl rubroST**	**0.274**	**0.036**
MI-leg	il rubroST	0.133	0.092	MI-leg	il rubroST	0.088	0.187	**MI-leg**	**il rubroST**	**0.218**	**0.031**

Supplementary Table 4: Linear regression results when combining gFA derived from one-directional voxels of the ipsilesional CST with gFA derived from two-directional extrapyramidal voxels. The results suggest that ipsilesional CST and extrapyramidal tracts were largely independent with respect to the explanation of behavioral variance in motor impairment. *(il = ipsilesional, cl = contralesional, CST = corticospinal tract, reticuloST = reticulospinal tract, rubroST = rubrospinal tract)*

DV	predictor 1	R^2	p	predictor 2	R^2	p
MI-arm	il CST	31.94%	0.003	cl rubroSt	51.02%	0.0004
MI-leg	il CST	17.71%	0.036	il reticuloST	38.86%	0.0045
MI-leg	il CST	17.71%	0.036	cl reticuloST	39.61%	0.0039
MI-leg	il CST	17.71%	0.036	il rubroST	38.94%	0.0044
MI-leg	il CST	17.71%	0.036	cl rubroST	39.85%	0.0037

Supplementary Figures

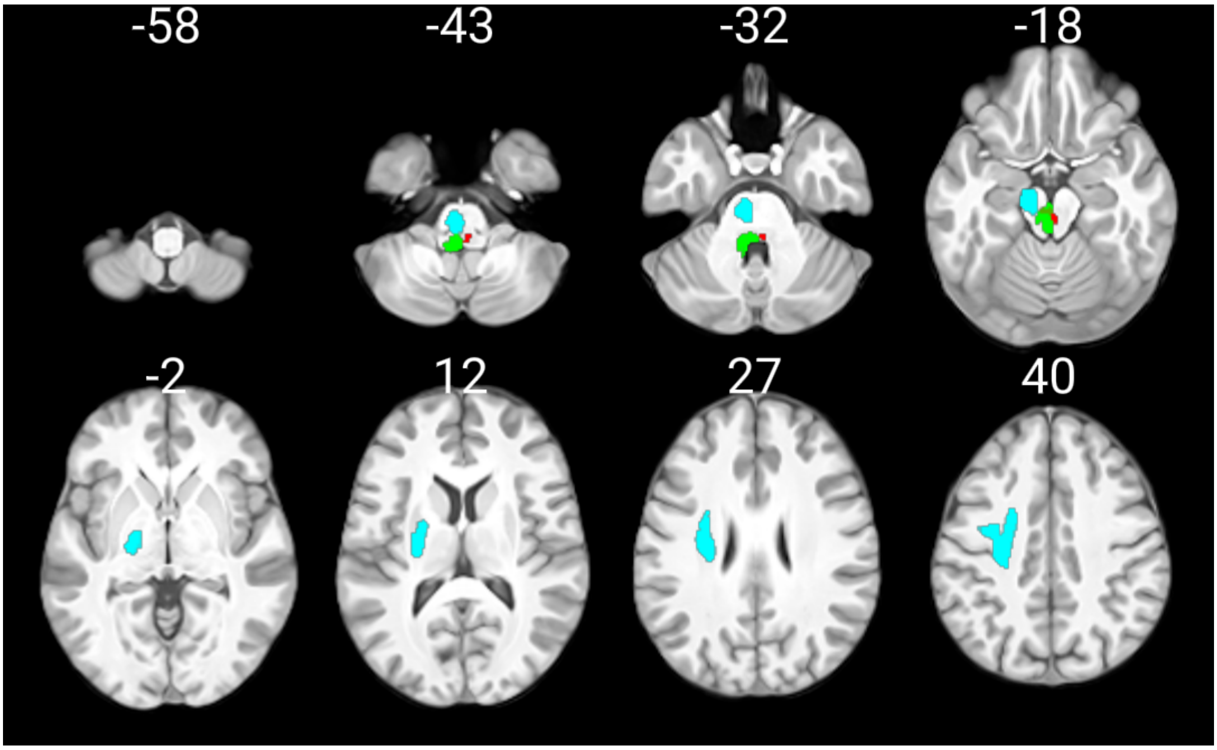

Supplementary Figure 1: Slicewise depiction of motor tracts descending from the left hemisphere. (*blue = corticospinal tract, green = reticulospinal tract, red = rubrospinal tract*)

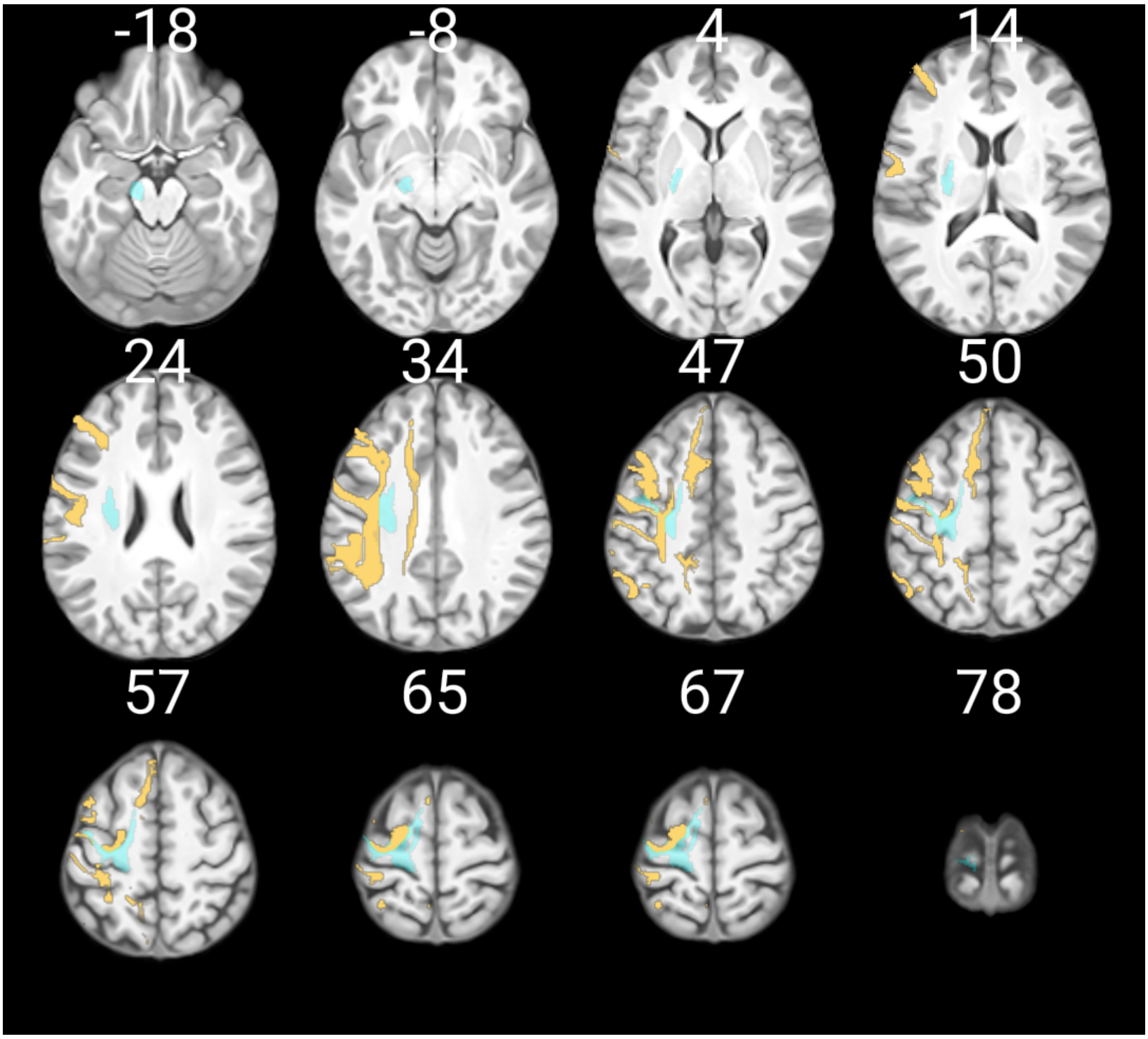

Supplementary Figure 2: Slicewise depiction of superior longitudinal fasciculus (SLF, *yellow*) and corticospinal tract (CST, *blue*). To exclude a possible bias of our findings introduced by accidentally sampling SLF voxels when extracting gFA from the CST, axial slices were assessed for a potential overlap of both tracts. As depicted in this figure, there was no considerable overlap between both tracts.

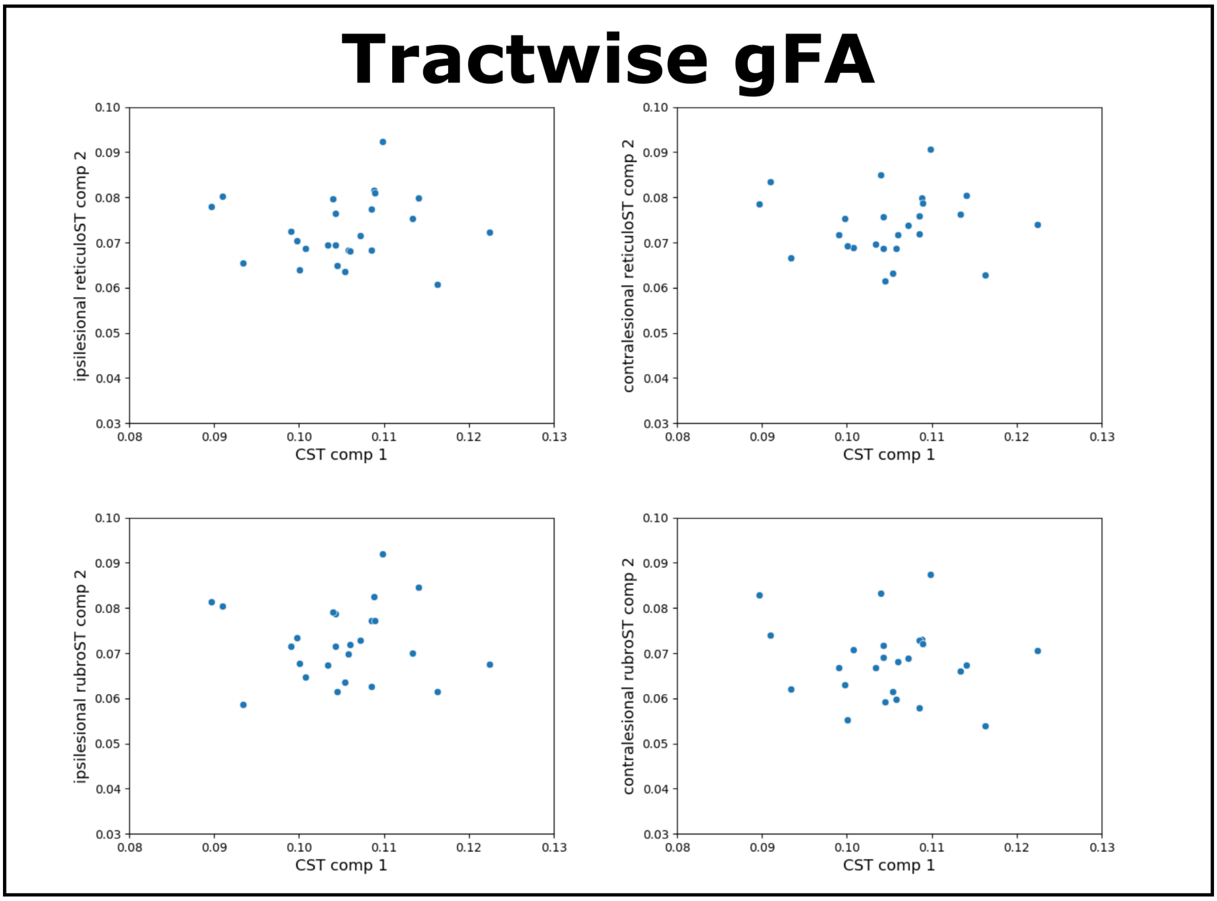

Supplementary Figure 3: Scatter plots of mean gFA derived from compartment 1 of the ipsilesional CST and compartment 2 of extrapyramidal tracts. The scatter plots indicate no correlation between those variables, ruling out multicollinearity between predictor variables as a potential bias that might hinder the interpretation of multiple linear regression models.
